# Do Technical Aids for Patient Handling Prevent Musculoskeletal Complaints in Health Care Workers?—A Systematic Review of Intervention Studies

**DOI:** 10.3390/ijerph15030476

**Published:** 2018-03-09

**Authors:** Janice Hegewald, Wera Berge, Philipp Heinrich, Ronny Staudte, Alice Freiberg, Julia Scharfe, Maria Girbig, Albert Nienhaus, Andreas Seidler

**Affiliations:** 1 Institute and Policlinic of Occupational and Social Medicine, Faculty of Medicine, Technische Universität Dresden, Fetscherstr. 74, 01307 Dresden, Germany; weraberge@live.de (W.B.); ph.heinrich.1986@gmail.com (P.H.); ronny.staudte@gmx.de (R.S.); alice.freiberg@tu-dresden.de (A.F.); julia.scharfe@gmx.de (J.S.); maria.girbig@tu-dresden.de (M.G.); andreas.seidler@mailbox.tu-dresden.de (A.S.); 2 Institute for Health Service Research in Dermatology and Nursing, University Clinics Hamburg Eppendorf, Martinistr. 52, 20246 Hamburg, Germany; Albert.Nienhaus@bgw-online.de; 3 Department of Occupational Health Research, German Social Accident Insurance Institution for the Health and Welfare Service, Pappelallee 33-37, 22089 Hamburg, Germany

**Keywords:** moving and lifting patients, musculoskeletal diseases, low back pain (LBP), occupational medicine, equipment and supplies, hospital, ergonomics, systematic review

## Abstract

The physical load ensuing from the repositioning and moving of patients puts health care workers at risk of musculoskeletal complaints. Technical equipment developed to aid with patient handling should reduce physical strain and workload; however, the efficacy of these aids in preventing musculoskeletal disorders and complaints is still unclear. A systematic review of controlled intervention studies was conducted to examine if the risk of musculoskeletal complaints and disorders is reduced by technical patient handling equipment. MEDLINE^®^/PubMed^®^, EMBASE^®^, Allied and Complementary Medicine Database (AMED), and Cumulative Index of Nursing and Allied Health Literature (CINAHL^®^) were searched using terms for nursing, caregiving, technical aids, musculoskeletal injuries, and complaints. Randomized controlled trials and controlled before-after studies of interventions including technical patient handling equipment were included. The titles and abstracts of 9554 publications and 97 full-texts were screened by two reviewers. The qualitative synthesis included one randomized controlled trial (RCT) and ten controlled before-after studies. A meta-analysis of four studies resulted in a pooled risk ratio for musculoskeletal injury claims (post-intervention) of 0.78 (95% confidence interval 0.68–0.90). Overall, the methodological quality of the studies was poor and the results often based on administrative injury claim data, introducing potential selection bias. Interventions with technical patient handling aids appear to prevent musculoskeletal complaints, but the certainty of the evidence according to GRADE approach ranged from low to very low.

## 1. Introduction

Numerous studies report a risk of occupational back complaints due to lumbar disc disorders, sprains and strains among health care workers [1,2,3,4,5,6,7,8,9,10]. The U.S. incidence rates in 2015 for nonfatal occupational injuries and illnesses caused by sprains, strains, and disc tears were 18.7 per 1000 for nursing assistants, and thereby higher than for emergency medical technicians and paramedics (17.8), laborers and freight, stock and material movers (12.0), metal workers (11.7), and construction workers (7.2), and only surpassed by firefighters (21.3) [11]. A systematic review examining the prevalence of musculoskeletal disorders in health care workers found that the one-year prevalence ranged from 28–96% [12].

A main reason for health care workers’ risk of musculoskeletal disorders is the manual lifting and transferring of patients, which places stress on the ligaments of the spine, especially the lumbar spine [10,13,14]. The lower back, the cervical spine and shoulder joints appear to be the most affected body parts [15,16,17]. Cumulative spinal loads due to occupational manual patient handling and forward bending working postures are associated with lumbar disk herniation [18,19,20] and lumbar degenerative disorders [19,21,22]. Also, the risk for lumbar disc disorders due to physical occupational exposures, such as manual lifting, are higher than for low back pain (LBP) alone, establishing physical workload as a risk factor for structural disc damage [20]. Other risk factors for musculoskeletal complaints among health care workers include sex, age (younger), work setting, and psychosocial factors like low job control [12].

Lumbar disc disorders and complaints result in substantial medical expenses and productivity loss in workers [23,24,25,26,27], and in several countries—such as Germany, France, Belgium, and Italy—lower back disorders can be considered occupational diseases [28]. The individual burden of LBP is also great, as Seidler et al. [29] show in their health utilities investigation of chronic LBP in a population of health care workers, where LBP patients would choose a 7% shorter life expectancy to avoid chronic LBP and healthy participants would even spend 10% of their life expectancy to avoid chronic LBP.

Freiberg et al. [30] examined the effectiveness of using small (non-technical) aids (e.g., sliding sheets and walking belts) during patient handling and their impact on the occurrence of musculoskeletal complaints with a systematic review, and found the current level of evidence for their preventive use to be inadequate. While evidence for the preventive use of small aids is deficient, until now the evidence from interventions with technical aids has not yet been considered in a systematic review.

Technical patient handling aids, also known as mechanical assistive devices, can reduce the number of manual patient lifts required. The resulting decrease in lifting forces experienced by health care workers during patient handling can help to prevent complaints and disorders due to overloading [31,32,33,34]. Therefore, the German Social Accident Insurance Institution for the Health and Welfare Service (Berufsgenossenschaft für Gesundheitsdienst und Wohlfahrtspflege (BGW)) [35] recommends the use of patient lifting devices to prevent physical workload while handling patients. Holtermann et al. [34] observed a possible benefit of technical aids in a prospective cohort study, where the odds ratio (OR) for developing infrequent LBP in female health care workers who occasionally used assistive devices during patient handling, compared with those who used them often, was 1.21 (95% confidence interval (CI) 0.90–1.62); for those who used them rarely, the OR was 1.78 (95% CI 1.19–2.66). 

The aim of this research was to review the evidence regarding the effect of technical patient handling devices on preventing complaints and disorders, including acute presentations of debilitation and pain (i.e., injuries), to the lower back, upper back, and the shoulder joints among health care workers. In contrast to small aids [30], technical patient handling devices were considered to be electrically powered motorized equipment that can be used as an alternative to manual lifting for transferring patients from one location to another or for re-/positioning patients in bed (e.g., nursing beds, low nursing home beds, bed movers, mobile lifts, wall lifts and overhead ceiling lifts, height adjustable baths).

## 2. Materials and Methods

A comprehensive search of the medical literature was performed to identify all controlled intervention studies examining whether providing technical patient handling aids to health care workers prevents musculoskeletal complaints and disorders. The review research question and subsequent study inclusion criteria were defined according to the PICO framework [36] as follows: a population (P) of health care workers, therapists, health care volunteers, and caregiving relatives between 15 and 70 years of age conducting patient handling and transfers, provided with technical patient handling equipment as an intervention (I) in comparison (C) to similar populations lacking equivalent equipment, in order to determine the impact of the technical patient handling equipment on the risk of complaints (including pain), disorders, or injuries to the upper or lower back and shoulder joints as outcomes (O) (Table 1). Perceived exertion and perceived risk of injury were excluded as outcomes. Since the aim of this study was to assess the evidence from controlled intervention studies, such as randomized controlled trials (RCTs) or controlled before-after studies (CBAs) (i.e., quasi-experimental studies with an intervention group and a non-randomized concurrently assessed control group), editorials, commentaries, narrative and systematic reviews, case–control studies, cross-sectional studies, expert opinions, case reports, and case series studies were excluded. Methodological details of this review are also available online in the a priori defined study protocol registered in PROSPERO (PROSPERO Registration: CRD42016029721).

### 2.1. Literature Indentification

The databases MEDLINE^®^ (via PubMed^®^ and OVID^®^), EMBASE^®^ (via OVID^®^), Allied and Complementary Medicine Database (AMED via OVID^®^), and Cumulative Index of Nursing and Allied Health Literature (CINAHL^®^ via EbscoHost^®^) were searched up to 16 February 2018 using search strings with keywords corresponding to the research question. The PubMed search strategy is available as an online supplement (see the Supplementary Materials Table S1). Reference lists of the included studies and key articles [37,38,39,40,41,42] were examined for further studies. Studies in all languages were included. Grey literature was not explicitly excluded or sought; however, publications consisting only of an abstract (i.e., conference proceedings) were excluded.

### 2.2. Screening

The selection of relevant studies based on titles and abstracts was conducted independently by two reviewers (Julia Scharfe and Janice Hegewald) and conflicts reconciled by a third reviewer (Alice Freiberg). The full-texts of the studies were also assessed for inclusion independently by two reviewers (Wera Berge/Alice Freiberg and Ronny Staudte/Janice Hegewald) and disagreements discussed until resolved.

### 2.3. Data Extraction and Quality Assessment

Data extraction was conducted by one reviewer (Wera Berge/Alice Freiberg) and the accuracy and completeness verified by a second reviewer (Janice Hegewald). The methodological quality of RCTs was assessed using the Cochrane risk of bias [36]. In accordance to the systematic review by Freiberg et al. [30], quasi-experimental CBAs were assessed with the questions for internal validity (questions 14–26) from the “Downs and Black checklist” [43], but using the risk of bias ratings: high risk, low risk, or unclear risk of bias instead of the recommended “0/1-rating”. Two questions pertaining to randomization (questions 23 and 24) were omitted, since non-randomized experimental study designs can generally be considered to have a higher risk of bias compared to randomized studies. In some cases, risk of bias judgements were adapted to better suit to the study question. For example, classical blinding of subjects is practically unachievable with this form of intervention, so studies that mention preventing interaction between the intervention and control groups were considered to have a low risk of bias for participant blinding, since at least subjects in the control group should have been unaware of the study’s aims. Similarly, interventions conducted in separate facilities from the comparison group, but not explicitly mentioning preventing study group interaction, were considered to have an unclear risk of bias regarding blinding of subjects. Two researchers (Wera Berge/Alice Freiberg and Janice Hegewald) assessed the methodological quality of the studies using these instruments and divergent assessments were discussed until consensus could be reached or mediated by a third reviewer (Andreas Seidler).

### 2.4. Data Synthesis and Evaluation of Evidence

Statistical synthesis of the resulting data (random-effects meta-analysis) was conducted with Review Manager (version 5.3) (The Cochrane Collaboration, Copenhagen, Denmark) when at least two of the studies considered similar outcomes with comparable/combinable effect estimates [44]. Finally, a summarizing evaluation of the evidence for the main outcomes was conducted in accordance with the GRADE method [36,45] using the GRADEpro Guideline Development Tool (Evidence Prime, Inc., Hamilton, ON, Canada).

## 3. Results

**Figure 1:**
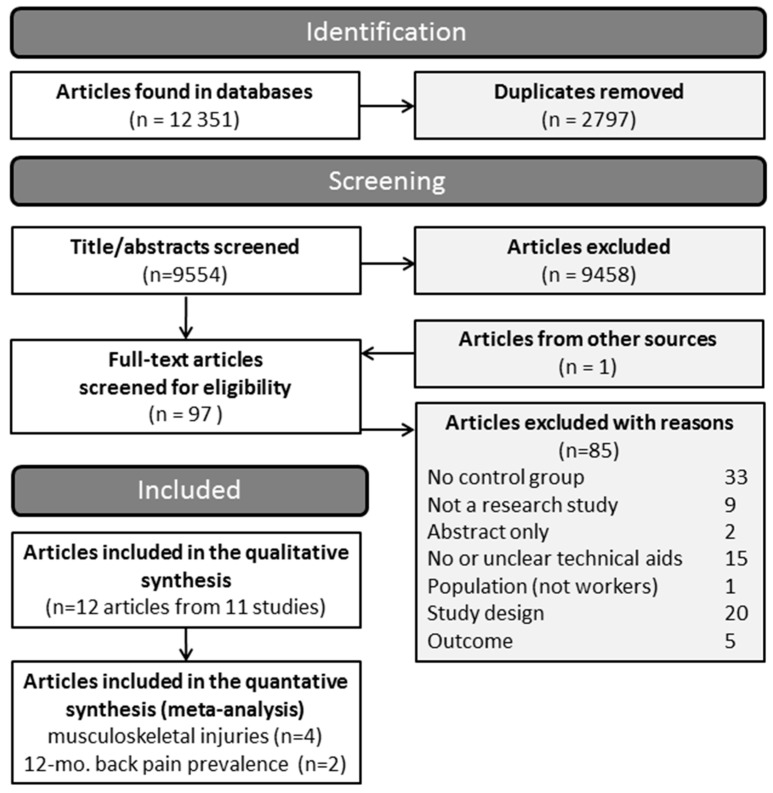
Flowchart depicting the literature identification process.

After the removal of duplicates, the search for articles resulted in the screening of 9554 titles and abstracts and 97 full-texts. A flowchart depicting the process of literature identification and reasons for the exclusion of full-texts is shown in Figure 1. The references of the excluded studies are listed in an online supplement (see the Supplementary Materials Table S2). The systematic search for literature resulted in 11 included studies from 12 articles published in 1999 to 2017 [46,47,48,49,50,51,52,53,54,55,56,57]. Ten of these studies were CBA studies [46,47,48,49,50,51,52,53,55,56] and one was an RCT [57]. Four of the studies were conducted in Canada, four in the USA, and one each in Italy, The Netherlands, and Great Britain.

Originally only studies of technical aids interventions compared to control groups without any technical aids were to be included (Table 1), this requirement was relaxed since several relevant studies were conducted in settings where at least limited technical patient handling equipment was available prior to the intervention. In these cases, the interventions involved increasing and improving the technical patient equipment available. Interventions were often multi-modal, encompassing educational sessions on the use of the new equipment and safe-patient handling, and sometimes implemented in conjunction with organizational changes, such as “safe lift” policies. A majority of the studies utilized already available administrative data of registered injury claims to examine changes in rates of patient-handling-related musculoskeletal injuries [47,48,49,50,51,53,54,55,57], and five studies examined changes in back pain prevalence [46,48,52,56,57]. Only three studies considered complaints and disorders to the neck or shoulder [47,48,57]. The characteristics of the included studies are summarized in Table 2 and the extracted results are shown in an online supplement (see the Supplementary Materials Table S3).

### 3.1. Acute Musculoskeletal Events (Injuries)

Six studies examined musculoskeletal injury rates based on administrative claims data without considering the anatomic locations afflicted, making this the most frequent outcome examined [47,49,50,51,55,57]. Often the studies examined musculoskeletal injury claims derived from registries of work-related injuries requiring medical treatment or resulting in lost work-time or restricted duties, which may have resulted in selection bias due to underreporting. Three of these studies only reported raw numbers of injuries observed during a certain period of time without providing the number of workers at risk [49,51,55], rendering the study evidence anecdotal at best. The extracted results of these studies are presented in an online supplement (see the Supplementary Materials Table S3).

#### 3.1.1. Quantitative Evidence

Of the three studies reporting injuries with (potentially) externally valid measurements (rates per full-time equivalents or 100,000 paid h) [50,54,57], only the study by Yassi et al. [57] was an RCT. This study implemented a three-arm, cluster-randomized design by randomizing nine units from three hospital areas (i.e., medical, surgical, and rehabilitation) to either a control arm (Arm A: “usual practice”) or one of two intervention arms (Arm B: “safe lifting” or Arm C: “no strenuous lift”). Besides using a study design with a greater level of evidence, this study also assessed musculoskeletal complaints and pain with validated questionnaires. Compared to the three-year average prior to the intervention, rates of all injury claims increased in the control group (Arm A) one year after the intervention (from 5.1 to 7.6 per 100,000 paid h). In comparison, the same rates declined in the “safe lifting” group (Arm B) from 6.3 to 5.3 per 100,000 paid h, and in the “no strenuous lift” group (Arm C) from 9.3 to 6.1 per 100,000 paid h. The authors did not report any results of statistical tests to determine if these differences were statistically significant. Also, it is not possible to differentiate the preventive effect of technical patient handling aids from that of the small aids.

Two studies examined the effect of distributing portable technical lifting equipment to nursing, acute care hospital units, and long-term care (LTC) units on the musculoskeletal injury claim rates using the rates among all workers not provided with technical patient handling equipment over the same time period to control for temporal trends and to estimate what the authors called adjusted rate ratios (RR) [50,53]. Evanoff et al. [50] found a post-intervention RR for musculoskeletal injury claim of 0.82 (95% CI 0.68–1.00) for all units combined, 0.86 (95% CI 0.69–1.08) for acute care units, and 0.71 (95% CI 0.49–1.03) for LTC units, and Li et al. [53] reported an RR of 0.50 (95% CI 0.20–1.26) for nursing units.

Two further CBAs examined the effect of multimodal interventions [47,48]. The study described in the publications by Black et al. [47] and Lim et al. [54] assessed an intervention that included provision of mechanical lifts to high risk units of the three intervention hospitals. The incidence of musculoskeletal injuries occurring during a patient handling maneuver (based on claims data) prior to and following the intervention were examined using Poisson regression. The estimated RR of musculoskeletal injuries post- vs. pre-intervention was 0.69 (95% CI 0.60–0.80) after adjusting for intervention group allocation and hospital size, and RR = 1.42 (95% CI 1.23–1.64) for the intervention vs. control group after adjusting for intervention period and hospital size (which most likely reflects the higher injury rate of the intervention group prior to the intervention) [47]. The more recent study by Dennerlein, et al. [48] also estimated the post- vs. pre-intervention injury claim RR with Poisson regression (RR = 0.87 (95% CI 0.76–1.00)).

**Figure 2:**
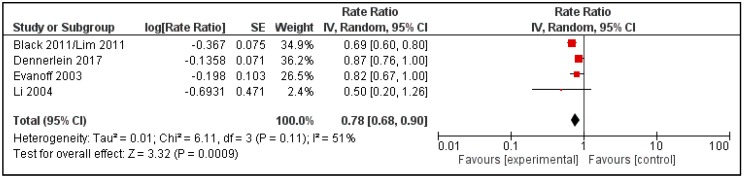
Forest plot of studies reporting the relative risk estimates for all musculoskeletal injuries in intervention versus control groups post-intervention.

The RR estimates for the outcome of acute musculoskeletal events (injury claims) post- vs. pre-intervention reported by four of the studies [47,48,50,53] were similar enough to be combined in a meta-analysis (Figure 2). The meta-analysis resulted in pooled RR of 0.78 (95% CI 0.68–0.90).

#### 3.1.2. Repeated Musculoskeletal Injuries

Only Lim et al. [54] examined a sub-set of employees with previous musculoskeletal injury claims to determine the effect of their intervention on the incidence of recurring injuries. They report a lower incidence of repeated back injuries (including neck, mid- and low-back) in the intervention group (21%) versus the control group (32%). Repeated shoulder injuries were slightly lower in the intervention group (11% vs. 14%), and no notable difference in neck injures was observed. A logistic regression model adjusting for sex, age, occupation type, work department, hospital size, and body part injured found that the intervention group had a lower odds of repeated musculoskeletal injuries compared to the control group (odds ratio, OR = 0.62; 95% CI 0.27–0.81).

### 3.2. Back Pain

Prevalence of back pain or low back pain was examined by five studies [46,48,52,56,57], but only the studies by Knibbe and Friele [52] and Baldasseroni et al. [46] described the impact of technical aid interventions on the 12-month prevalence of back pain. 

Yassi et al. [57] examined low back pain using both one-week prevalence of work-related low-back pain ratings and Oswestry Low-Back Pain Disability scores. The average Oswestry scores were slightly lower at the 12-month follow-up compared to baseline in both intervention groups (Arms B and C) and slightly increased in the control arm (Arm A) (not statistically significant). Only the “safe lifting” group (Arm B) with an intervention emphasizing small aids had a significantly lower one-week prevalence of work-related low-back pain at the 6- and 12-month follow-ups.

Dennerlein et al. [48] assessed the impact of the intervention on the three-month prevalence of back pain and musculoskeletal pain severity using adapted versions of the NordicQ and Disabilities of the Arm, Shoulder and Hand (DASH) instruments, respectively. The post- vs. pre-intervention adjusted OR for the three-month prevalence of back pain was 0.81 (95% CI 0.63–1.04) and 0.96 (95% CI 0.74–1.24) for moderate musculoskeletal pain severity.

Smedley et al. [56] assessed the one-month prevalence of low back pain with questionnaires mailed to the nursing staff of two hospitals in southern England before and after implementation of a multimodal intervention at one of the hospitals (intervention started 18–28 months after baseline survey and the follow-up survey was conducted 32 months after baseline survey—implying the intervention period varied from 4 to 14 months). The one-month prevalence of low back pain increased slightly in the intervention group at the follow-up assessment (not statistically significant) and remained unchanged at the control hospital. The number of patient-handling activities reported at both hospitals remained largely unchanged, with even a slight decrease in activities observed for the control hospital. Smedley et al. [56] suggest that this decrease might have been due to the unanticipated and undocumented efforts to improve manual-handling training and increase the use of patient handling equipment implemented by the management of the control hospital during the study period. 

The study by Knibbe and Friele [52] was the oldest study found and the only study to examine the effectiveness technical aids among home care nurses. The number of patient lifts conducted pre- and post-intervention was also assessed in a sample of the study population (intervention: *n* = 50; control: *n* = 54) using a lift counter (LC) log. The total average number of lifts conducted was reduced in the intervention group from 35 to 21 after the introduction of the patient hoists, while the total average number of lifts in the control group remained stable at 24. The 12-month prevalence of back pain could also be significantly reduced in the intervention group from 74 to 64%, while the prevalence in the control group increased slightly from 62 to 66%. The authors acknowledge that the observed pre-intervention differences were probably due to unintended selection, and speculate that the post-intervention reduction in lifts logged were the result of family members assuming some of the required lifting tasks in the absence of the home care nurses with the help from the hoists.

Baldasseroni et al. [46] gradually distributed equipment to departments according to their assessed needs, resulting in an unknown amount of variation in the length of the intervention and follow-up. The 12-month prevalence of low back pain for the health care workers provided with patient equipment was 31% pre- and 11% post-intervention compared to 24% and 21% in the control group, respectively. The results of this study also do not permit differentiation effects due to small aids and technical patient handling aids.

**Figure 3:**
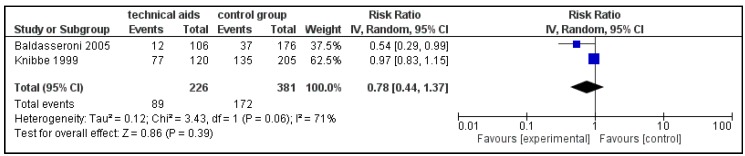
Forest plot of post-intervention 12-month prevalence rate ratios for back-pain in intervention vs. control (reference) groups.

The pooled post-intervention prevalence ratios indicated the effectiveness of the intervention but did not reach statistical significance (Figure 3). In addition, the results of the two studies were heterogenous. It also is worth noting that the pre-intervention 12-month back pain prevalence was lower in the control groups of both studies compared to the intervention groups. This was probably due to the selection of the intervention groups based on their equipment needs. Considering that the groups were not comparable at baseline, the pooled post-intervention risk ratio may underestimate the actual effect of the intervention on the prevalence of pain. However it is also possible that the preventive impact of such interventions is greater in workplaces with increased needs. 

### 3.3. Cervical Spine

Two CBAs examined the pain and injury claims for the cervical spine [47,48]. Black et al. [47] found the percentage of neck injury claims observed in both the intervention (IG) and control groups (CG) increased post-intervention, however the increase was greater in the control group (pre-intervention: IG = 4.6%; CG = 8.4% vs. post-intervention: IG = 6.6%; CG = 14.5%). The affiliated publication by Lim et al. [54] considering the impact of the intervention on repeat injuries found no difference in the risk of repeat injuries to the cervical spine between study groups. Dennerlein et al. [48] estimated adjusted ORs for the three-month prevalence of neck/shoulder pain (post- vs. pre-intervention) of 0.90 (95% CI 0.70–1.16) and 0.91 (95% CI 0.77–1.08) for the intervention and control groups, respectively. The reduced risk of neck/shoulder injury claim RRs for this study were 0.678 (95% CI 0.46–1.00) in the intervention group and 0.713 (95% CI 0.33–1.55) in the control group.

### 3.4. Shoulder

Only Yassi et al. [57] examined shoulder pain using the DASH score and one-week prevalence of work-related shoulder pain ratings. DASH scores were slightly lower at the 12-month follow-up compared to baseline in both intervention groups (Arms B and C), and slightly increased in the control arm (Arm A) (not statistically significant). Only the “safe lifting” group (Arm B) had a significantly lower one-week prevalence of work-related shoulder pain at the 6- and 12-month follow-ups.

Black et al. [47] found the percent of injury claims for the shoulder increased in both study groups, but the increase was more pronounced in the control group. The same study found a higher percentage of repeated shoulder injuries in the control group (14%) compared to the intervention group (11%) [54].

### 3.5. Risk of Bias and GRADE Assessments

The results of the risk of bias assessments with the “Downs and Black” and the Cochrane risk of bias are summarized in an online supplement (See the Supplementary Materials Table S4). All of the studies had at least one serious methodological limitation that raised concerns regarding bias, so the overall within-study risk of bias of all of the studies was judged to be “high”.

The methodological quality of the studies contributed to the overall assessment of the body of evidence for each of the outcomes. The evidence was examined with the GRADE approach for what were considered to be important outcomes using the GRADEpro software [58]. The selected outcomes were restricted to those providing the most evidence regarding the effectiveness of technical patient handling aid interventions. The outcomes considered were acute musculoskeletal events (based on injury claims), 12-month prevalence of (low-) back pain, cervical spine injuries, shoulder pain, and repeated musculoskeletal injuries. The body of evidence for each of these outcomes was determined to be “low” to “very low” as depicted in the summary of findings table (Table 3).

## 4. Discussion

Overall the results of the studies seem to indicate that interventions with technical patient handling aids may help prevent musculoskeletal complaints and disorders in health care workers. However, due to the very low quality of the available evidence, this preventive effect is uncertain. Although Freiberg et al. [30] found no convincing evidence that interventions with small aids, such as sliding sheets, can prevent musculoskeletal outcomes, it seemed reasonable to expect stronger effects from interventions with technical aids. However, this was only partially true. Although more studies examining the impact of technical aids were found, the limited methodological quality of the studies render the moderate effects observed unconvincing. In addition, some of the intervention studies included the provision of small aids with the intervention, making it impossible to determine how much of the preventive effect was can be attributed to the use of technical aids. One study excluded small aids from the intervention [48] and seven studies did not explicitly mention the inclusion of small aids in the intervention [47,49,50,51,52,53,55], but only five of these studies presented the results in an interpretable form. These studies found a reduction in 12-month back pain prevalence [48,52], and reduced injury rates in the following the intervention [47,48,50,53].

The non-randomized studies often reported selecting “at risk” units to receive the intervention, obscuring the results of the assessment. This risk imbalance could have resulted in an overestimation of the effect due to the greater potential impact of the interventions in “at risk” units. However, if the impact of the intervention was not dependent on the pre-existing increased risk, the post-intervention RRs may underestimate the potential effect of the intervention. In other words, the intervention brought the increased pre-intervention back pain prevalence in the intervention groups to levels similar to, yet slightly lower than that of the control group, resulting in a post-intervention RR closer to the null value. Thus the observed effect might have been greater if the groups had been comparable from the start.

Two of the studies appeared to have used planned renovations as an opportunity to examine the impact of ceiling lifts on patient handling practices and injury claims [49,55]. However, these studies reported absolute numbers of injury claims without taking the number of employees at risk or their characteristics (i.e., age, sex) into account. Consequently, the validity of these results is questionable, and the uncertainty of the evidence increased by poor or incomplete reporting.

Unfortunately, none of the studies used medical evaluations or physician diagnosed disorders as outcomes. Such information could have provided objective insights regarding the progression of musculoskeletal degeneration following interventions. A number of studies examined self-reported complaints (i.e., pain), for the most part with validated and widely used instruments. However, most of the studies evaluated injury claims data as a form of secondary data analysis, since these data were readily available. Although this registry data was less likely to be biased due to the lack of outcome assessor blinding, injury claims may be subject to selection bias. Some workers may choose not to report their injuries, and some claims require a long time to process and might have been overlooked. While musculoskeletal injuries can result from a single traumatic incident, an accumulation of subclinical trauma over many years places workers at risk for acute presentations of incapacitating pain. Longer follow-up times and differentiated examination of injury types could provide more convincing evidence regarding the impact of technical aids on musculoskeletal injuries.

Prevalence of back pain was assessed by four studies, with three studies specifically examining low-back pain [46,48,56], while Knibbe and Friele [52] reported the prevalence of back pain (not further specified). Nevertheless, the prevalence of low-back and back pain were considered together in the qualitative synthesis and the pooling of risk estimates, but it is unclear if this difference in outcome definition may have had any noteworthy impact on the overall results.

Several papers attempted to assess compliance with the intervention or actual use of the equipment with partially contradictory results. For example, Miller et al. [55] and Engst et al. [49] found 71–75% of the intervention unit workers surveyed reported the new ceiling lifts were their preferred method of patient transfer. Dennerlein et al. [48] found the self-reported safe handling practices were significantly increased in the intervention group post-intervention (*p* < 0.005) while unchanged in the control group. Yassi et al. [57] found the self-reported use of technical aids for patient lifting peaked in both intervention arms at the six-month follow-up, but declined some by the 12-month follow-up. Li et al. [53] outfitted the lifts with mechanical counters to examine compliance, and report that the initial rate of lift use was lower than expected and sank even lower after three months. Evanoff et al. [50] conducted interviews with a convenience sample of employees in the intervention units regarding the use of lifting equipment during the last shift and found the self-reported use of lifts was higher in LTC units and very low among registered nurses (6.36%) compared to other the other health care workers (38.75%). These results suggest that continual and directed efforts may be needed to facilitate or encourage equipment use.

No evaluation of publication bias was conducted, because so few studies could be considered in the meta-analyses. Therefore, we cannot rule out that publication bias may exist for studies of technical aid interventions. Unfortunately, it is questionable whether online registries for clinical trials would be used to report planned ergonomic intervention studies.

To the best of our knowledge this is the first systematic review to consider evidence regarding the effectiveness of technical patient handling aid intervention studies. This review intended to include and consider all available medical literature meeting the a priori inclusion criteria. Results of studies were summarized with meta-analyses when possible, and the strength of the body of evidence was assessed by considering the risk of bias of the individual studies and by applying the GRADE approach.

As a consequence of the very low level of existing evidence, quality research is still needed. However, this evidence may need to come from high quality observational studies, as technical aids are already widely available in workplaces where frequent patient handling is required. It would be impractical, if not unethical, to withhold such equipment from employees in order to conduct controlled experimental studies.

## 5. Conclusions

Interventions with technical patient handling aids indicate that this equipment may help prevent musculoskeletal complaints and disorders, but the certainty of the evidence according to GRADE ranged from low to very low. However, the wide-spread availability of technical patient handling aids will make future intervention studies problematic.

## Figures and Tables

**Table 1:** Study inclusion criteria according to the PICO framework.

PICO	Study Inclusion Criteria
Participants (P)	Labor force from the field of nursing exerting patient transfers; especially health care workers, therapists (physiotherapists, occupational therapists), as well as volunteer workers from the health care area, and caregiving relatives between the ages of 15 and 70 years.
Intervention (I)	Technical patient handling equipment (i.e., nursing beds, low nursing home beds, bed movers, mobile lifts, wall lifts, overhead lifts, ceiling lifts, day care chairs, or mechanical position change aids); as a solitary measure or as part of a multimodal intervention (e.g., combined with education, training, guidance, small assistive devices, etc.).
Comparison (C)	Health care and nursing settings lacking technical patient handling equipment.
Outcomes (O)	Complaints or disorders (self-reported disorders or pain assessed with any questionnaire e.g., Nordic, Disabilities of the Arm, Shoulder and Hand (DASH), Oswestry), including acute presentations of debilitation and pain ensuing in conjunction with a patient handling maneuver (i.e., injuries), in the (i) lumbar spine area; (ii) cervical spine area; or (iii) shoulder area.
Study design	Randomized controlled trials (RCT) and controlled before-after (CBA) studies

**Table 2:** Study characteristics.

Study, Year	Study Design (Follow-Up); Setting(s)	Participant Characteristics	Intervention(s)	Comparison	Outcomes
Baldasseroni 2005 [46]	CBA (1.5 years) five hospitals—Public Health Department Florence, Italy nursing professionals and health care workers involved in patient care	total employed at baseline *n* = 730 avg. age (at baseline) 38.7 years (±8.0) women 221 (78.1%) intervention group n_baseline_ = 167 n_follow-up_ = 136 loss to follow-up = 18.9% response after follow-up *n* = 106 missing response = 22.1% control group n_baseline_ = 563 n_follow-up_ = 297 loss to follow-up = 47.2% response after follow-up *n* = 176 missing response = 40.7%	total employed at baseline *n* = 730 avg. age (at baseline) 38.7 years (±8.0) women 221 (78.1%) intervention group n_baseline_ = 167 n_follow-up_ = 136 loss to follow-up = 18.9% response after follow-up *n* = 106 missing response = 22.1% control group n_baseline_ = 563 n_follow-up_ = 297 loss to follow-up = 47.2% response after follow-up *n* = 176 missing response = 40.7%	electromechanical lifts sliding lift sheets height-adjustable stretchers ergonomic lifting belts equipment varied depending on specific needs of the departments equipment was introduced over a 13-month period (between surveys)	no equipment provided	12-month prevalence of low-back pain (number of episode categories)
Black 2011/Lim 2011 [47,54]	CBA (two years) six hospitals in two health regions of Saskatchewan, Canada intervention group: hospital A: large, tertiary hospital with 436 beds hospital B: medium-sized community hospital with 239 beds hospital C: small hospital with long-term care (LTC) facility with 240 residents control group: three hospitals matched to intervention hospitals by hospital types and size	characteristics of injured workers at hospitals A, B, and C (pre-/post-intervention) [47]: intervention group (*n* = 260/*n* = 151) avg. age 40.5 years (±10.4)/41.0 years (±10.2) women 236 (91%)/142 (94%) control group (*n* = 139/*n* = 165) avg. age 39.2 years (±10.1)/39.1 years (±10.7) women 127 (91%)/161 (94%) employees with a previous patient handling-related injury at hospitals A, B and C (*n* = 1480) [54]: intervention group (*n* = 789) avg. age 41.2 years (±10.1) women 734 (93%) control group (*n* = 691) avg. age 39.3 years (±10.2) women 628 (91%)	Transfer, Lifting, and Repositioning (TLR) program • 2 mechanical lifts distributed to high needs units • eight-hour mandatory educational session (incl. anatomy, injuries, body mechanics, personal health, lifting and patient handling procedures, and patient-handling skills development) + yearly refresher (one hour) + course booklet and training materials program was introduced over a 10 or 12-month period	no injury prevention program, “standard occupational health and safety practice”	back injuries claims neck injury claims shoulder injury claims rate ratios musculoskeletal injury claims, post vs. pre-intervention repeated back injury claims repeated neck injury claims repeated shoulder injury claims odds ratio of repeated musculoskeletal injury claims
Dennerlein 2017 [48]	CBA (one year) two academic hospitals in the metropolitan area of Boston, Massachusetts (one intervention hospital, one control hospital)	random sample of employees in patient care units (with patient care duties) supervised by a nurse manager, employed in 2012, and working more than 20 h per week were surveyed: intervention group randomly selected *n* = 866 n_baseline_ = 580 women 528 (93.5%) avg. age 42.7 years (±0.49) n_follow-up_ = 499 (424 filled out both) response = 67.0% loss to follow-up 26.9% control group ^a^ randomly selected *n* = 1267 n_baseline_ = 1011 women ^a^ (91.4%) avg. age 40.6 years (±0.43) n_follow-up_ = 971 (785 filled out both) response = 79.8% loss to follow-up 22.4%	hospital-wide safe patient handling and mobilisation program comprising: • investment in ceiling lifts, slings, mechanical sit-to-stand devices, air-assisted lateral transfer devices, mobile lift devices and ceiling lifts in selected units (and no investment in small aids) • patient handling policy • program training (including mobility assessment training) • instructional bedside cards, instructions on mobile equipment, and “decision guides” distribute • new employees trained by a co-worker in a simulation laboratory • implementation of an equipment maintenance plan • information collected in a handbook for each unit • equipment needs assessments • patient mobility needs assessment • internal marketing campaign of the program • patient education material program was introduced over an eight month period	employees of four units in the comparison hospital were offered a well-being intervention unclear amount of technical equipment available (presumably similar to pre-intervention conditions at the intervention hospital)	three-month prevalence of low back pain (NordicQ) three-month prevalence of neck/shoulder pain (NordicQ) three-week intensity of musculoskeletal pain (adapted DASH upper limb score) back injury claim rate ratio (RR, 95% CI), post- vs. pre-intervention neck/shoulder injury claim rate ratio (RR, 95% CI), post- vs. pre-intervention injury claim rate ratio (RR, 95% CI), post- vs. pre-intervention
Engst 2005 [49]	CBA (one year) two 75-bed extended care units of a community hospital in British Columbia, Canada (one intervention unit, one control unit)	aged 19–60+ years sex not reported care aids, licensed practical nurses, registered nurses	• installation of ceiling lift tracks in 75-bed extended care unit • one-hour training session provided by on-site occupational therapist on use and care of ceiling lifts • a “no-unsafe manual lift policy” was developed and implemented ceiling lift renovations and training required six months	3 mechanical floor lifts, 1 sit-stander) were already available	raw number of claims for “lifting & transferring related injuries” and “repositioning related injuries”
Evanoff 2003 [50]	CBA (2–3 years) 31 intervention nursing units in St. Louis (Missouri), USA ^b^ (incl. neurology, orthopedics, intensive care, rehabilitation, general surgery, general medicine) from four acute care hospitals (1 major teaching hospital, 2 large suburban, 1 smaller community hospital) 5 LTC intervention units from 5 facilities in St. Louis (Missouri), USA (included 3 sites in St. Louis + 2 rural sites)	age and sex distribution of intervention and comparison groups not reported nurses, nursing aides, patient care technicians 190 health care workers in interventions units interviewed (with consent)	• 22 stand-up lifts (“EZ-Stand”) and 25 full-body lifts (“EZ-Lift”) distributed among the 36 intervention unit • two-hour hands on instructional course on lift operation Units received either both or only one lift (depending on the unit’s needs) time required to introduce equipment to all units was not reported	compared with injury data from all hospital workers not provided with new technical patient handling equipment at each facility during the same time frame	musculoskeletal injury claims rate ratio (RR, 95% CI), post- vs. pre-intervention
Fragala 2012 [51]	CBA “pilot study” (one year) 2 LTC units of the Radius Mayflower LTC facility in Plymouth (Massachusetts), USA (resident population had high level of dependency)	age and sex distribution of intervention and comparison groups were not reported	“CASE program” ^c^ • 4 full sling lifts and 1 stand-assist lift following needs assessment • education and training sessions with vendor demonstration • pre- and post-intervention questionnaire time required to introduce equipment to all units not reported	no equipment provided	raw number of injury claims pre-/post-intervention
Knibbe & Friele 1999 [52]	CBA (one year) 20 teams of home care nurses working in Rotterdam, The Netherlands providing professional nursing care around the clock, seven days/week for patients living at home 8 intervention teams (*n* = 139) 12 control teams (*n* = 239)	avg. age 34.6 years (±8.8); range 21–58 years avg. work experience 13.3 years (±8.1); range 1–36 years avg. working hours 26.8 h/week (±11.7); range 2–49 h/week day-shifts 73.3%	• 40 patient hoists provided • training • ergonomic assessment forms • 12 specifically trained ‘lifting coordinators’ program was introduced over a 12-month period	no special equipment provided (two patient hoists already available)	12-month prevalence of back pain
Li 2004 [53]	CBA (seven months follow-up) three nursing units of a 111 bed community hospital in St. Louis (Missouri), USA (incl. medicine/surgery, intensive care, subacute care units)	health-care workers directly involved with patient handling intervention group 138 health care workers/nurses employed in the three units in 2000 n_baseline_ = 61 response = 44.2% n_follow-up_ = 36 loss to follow-up = 41.0% age and sex distribution of intervention and comparison groups not reported	• 1 portable full body sling lift • 2 portable stand-up sling lifts (“EZ-Lift” and “EZ-Stand”) • one time hands-on training sessions offered by hospital personnel at start of intervention equipment was introduced over a six month period	compared with injury data from units not provided with new technical patient handling equipment during the same time frame (mechanical lifts were not available)	musculoskeletal injury claim rate ratio (RR, 95% CI), post- vs. pre-intervention
Miller 2006 [55]	CBA (1 year) 2 LTC facilities in Vancouver (British Columbia), Canada intervention: 63-bed LTC facility (moved into newly constructed facility on same property on 1 August 2002) control: 100-bed LTC facility with similar patients and managed by the same organization	intervention (*n* = 45) responded to survey *n* = 17 women 94.1% age distribution 19–29 years 0% 30–39 years 41.2% 40–49 years 35.3% 50–59 years 11.8% 60+ years 5.9% missing: 5.9% control (*n* = 29) responded to survey *n* = 15 women 100% age distribution 19–29 years 0% 30–39 years 33.3% 40–49 years 46.7% 50–59 years 13.3% 60+ years 0% missing 6.7%	• ceiling lift tracking from beds to washrooms installed in all rooms; 6 portable ceiling lift motors purchased; 4 portable motors were purchased eight months post-intervention • one-hour training session with vendor demonstration on how to lift, transferring and repositioning patients one day was needed to move to the newly constructed facility	no ceiling-lift tracking both the intervention and control facilities had 4 mechanical lifts prior to and during the intervention	raw number of injury claims pre-/post intervention
Smedley 2003 [56]	CBA (4–14 months ^d^) 2 National Health Service (NHS) hospitals in Southern England, UK providing acute medical and surgical services	baseline questionnaire ^e^ intervention (*n* = 817) response rate 54% age distribution <30 years 20% 30–39 years 36% 40–49 years 25% ≥50 years 19% part-time 45%/full-time 55% control group (*n* = 340) response rate 61% age distribution <30 years 20% 30–39 years 29% 40–49 years 30% ≥50 years 21% part-time 51%/full-time 49%	• manual-handling policy revised to “minimize unassisted patient handling and exposure to high-risk nursing tasks” • organizational engagement incl. managers from every level • 700 new sliding sheets for all wards • lifting and handling equipment, (incl. height adjustable baths, hoists, transfer belts, and sliding sheets) distributed to selected departments • “link nurses”-network: contact persons on ward; disseminates information to wards; responsible for equipment • two-day training course in health and safety offered; incl. basic aspects of manual handlingprogram was introduced over a ten month period.	“Limited” steps to improve manual-handling training and use of patient-handling equipment was initiated by the control hospital management during the study period.	one-month prevalence of low back pain
Yassi 2001 [57]	3-arm cluster RCT (one year) Winnipeg’s Health Sciences Centre in Winnipeg (Manitoba), Canada, an acute and tertiary care hospital nine units from medical, surgical, and rehabilitation service areas (three units/area) with high risk for musculoskeletal injury, similar patients, personnel, and size • each unit of a service area randomized to a study-arm • units were physically separate	346 nurses and unit assistants arm A *n* = 103/followed *n* = 82 (80%) arm B *n* = 116/followed *n* = 85 (73%) arm C *n* = 127/followed *n* = 94 (74%) age and sex distribution not reported	“Safe lifting” (Arm B) biomechanical strain reduced with small aids (e.g., transfer belts and slide devices) • 1 mechanical total body lift/unit • Transfer belt in each room • 2 large & 4 small sliding devices/unit “no strenuous lift” (Arm C) eliminate/reduce patient transfers with help from technical devices • mechanical total body lifts ^f^ • sit-stand lifts ^f^ • 1 large & 2 small sliding devices/roomboth arms received: • 3-h hands-on education: back care, patient assessment, transfer techniques, use of transfer aid equipment time required to introduce the program was not reported	“Usual practice” (Arm A) • biomechanics & lifting techniques training on request • training for equipment already in use • 1 mechanical total body lift/ward • sliding devices (on request)	musculoskeletal injury claim rate per 100,000 paid h one-week prevalence of work-related low-back pain ratings Oswestry back disability scores one-week prevalence of work-related shoulder pain ratings DASH upper limb score (includes arm, shoulder, and hand)

^a^ Numbers reported in publication were weighted due to oversampling. ^b^ “Intervention units were chosen based on … past injury rates, the expressed interest of nursing management, and perceived risk of injuries posed by different patient populations” [50]; ^c^ The CASE program is described as a five-step framework beginning with identification of high-risk activities and resident assessment. Intervention unit was selected based on past injury rates [51]; ^d^ Intervention began 18–28 months after baseline survey, and follow-up survey was 32 months after baseline [56]. ^e^ Study was restricted to women [56]. ^f^ Number of lifts provided depended on the unit’s patient population [57].

**Table 3:** Summary of findings.

Outcomes	No. of Participants (Studies) Follow-Up	Certainty of the Evidence (GRADE)	Relative Effect (95% CI)	Anticipated Absolute Effects	
				Risk with Few or no Patient Transferring Aids	Risk Difference with Technical Aids (Range Based on 95% CI)
musculoskeletal injuries (no site differentiation) assessed with: claims data follow up: range 1 to 2 years	NR (4 observational studies)	⨁**◯◯◯** VERY LOW ^a^	RR 0.78 (0.68 to 0.90)	Not computable due to unreported number of workers at risk in the studies	
12-month prevalence of (low-)back pain assessed with: post-intervention RR follow up: range 1 to 2 years	607 (2 observational studies)	⨁**◯◯◯** VERY LOW ^b,c^	RR 0.78 (0.44 to 1.37)	45 per 100	10 fewer per 100 (25 fewer to 17 more)
repeated musculoskeletal injuries assessed with: claims follow up: 2 years	1480 (1 observational study)	⨁**◯◯◯** VERY LOW ^a^	OR 0.62 (0.27 to 0.81)	22 per 100	7 fewer per 100 (15 fewer to 3 fewer)
cervical spine (neck) injuries assessed with: injury claims follow up: range 1 to 2 years	1786 (2 observational study)	⨁**◯◯◯** VERY LOW ^a^	One study found the percentage of injury claims involving the neck increased post-intervention in both the group receiving the intervention as well as the control group. The other study found the a nearly statistically significant protective post-intervention RR for neck/shoulder injury claims		
shoulder pain assessed with: 1-week shoulder pain rating follow up: 1 year	261 (1 RCT)	⨁⨁**◯◯** LOW ^d^	The RCT reported a reduction in 1-week shoulder pain ratings at the 6- and 12-month follow-ups in the intervention group receiving technical aids to prevent strenuous lifting. A similar reduction in pain rating was not observed in the control group or in the second intervention arm receiving primarily small aids. Although the 1-week prevalence of work-related shoulder pain at the 6- and 12-month follow-ups was significantly lower in the second intervention arm receiving primarily small aids		
The risk in the intervention group (and its 95% CI) is based on the assumed risk in the comparison group and the relative effect of the intervention (and its 95% CI) CI: Confidence interval; RR: Risk ratio; OR: Odds ratio; NR: not reported					
GRADE Working Group grades of evidence High certainty: We are very confident that the true effect lies close to that of the estimate of the effect Moderate certainty: We are moderately confident in the effect estimate: The true effect is likely to be close to the estimate of the effect, but there is a possibility that it is substantially different Low certainty: Our confidence in the effect estimate is limited: The true effect may be substantially different from the estimate of the effect Very low certainty: We have very little confidence in the effect estimate: The true effect is likely to be substantially different from the estimate of effect					

^a^ The study design was not randomized (controlled before and after study design). ^b^ The study design was not randomized (controlled before and after study design). One of the two studies reported significantly different prevalence rates prior to the intervention [52]. Both studies were subject to risk of bias due to lack of blinding [46,52]. ^c^ The confidence interval of the pooled risk estimate is wide. ^d^ The study participants and the researchers were not blinded.

## Supplementary Materials

The following are available online at http://www.mdpi.com/1660-4601/15/3/476/s1, Table S1: Pubmed search strategy, Table S2: excluded study references, Table S3: extracted results; Table S4: Risk of bias assessments.
