# Supplementary material for: Do Technical Aids for Patient Handling Prevent Musculoskeletal Complaints in Health Care Workers?—A Systematic Review of Intervention Studies
Source: Int J Environ Res Public Health. 2018 Mar 9;15(3):476. doi: 10.3390/ijerph15030476 (PMC5877021; doi:10.3390/ijerph15030476)
Supplement: Supplementary file 1 [file ijerph-15-00476-s001.docx]

**Table S1.** Pubmed search strategy

| #1 | Search ("moving and lifting patients"[MeSH Terms] OR (moving[tw] AND lifting[tw] AND patients[tw]) OR "moving and lifting patients"[tw] OR (patient[tw] AND handling[tw]) OR "patient handling"[tw] OR "patient transfer"[MeSH Terms] OR (patient[tw] AND transfer[tw]) OR "patient transfer"[tw] OR therapist[tw] OR "physical therapists"[MeSH Terms] OR (physical[tw] AND therapist[tw]) OR "physical therapist"[tw] OR physiotherapist[tw] OR "occupational therapist"[tw] OR (occupational[tw] AND therapist[tw]) OR "health personnel"[MeSH Terms] OR (health[tw] AND personnel[tw]) OR "health personnel"[tw] OR (healthcare[tw] AND workers[tw]) OR "healthcare workers"[tw] OR "nurses"[MeSH Terms] OR nurses[tw] OR nurse[tw] OR "nursing homes"[MeSH Terms] OR (nursing[tw] AND homes[tw]) OR "nursing homes"[tw] OR (nursing[tw] AND home[tw]) OR "nursing home"[tw] OR "retirement"[MeSH Terms] OR (retirement[tw] AND home[tw]) OR "homes for the aged"[MeSH Terms] OR (homes[tw] AND aged[tw]) OR "homes for the aged"[tw] OR (old[tw] AND age[tw] AND home[tw]) OR "old age home"[tw] OR (residential[tw] AND care[tw] AND home[tw]) OR caregiver[tw] OR ((family[tw] OR Family[MeSH Terms]) AND care[tw]) OR Caregivers[MeSH Terms] OR ambulatory[tw] OR ambulant[tw] OR "ambulatory care facilities"[MeSH Terms] OR (ambulatory[tw] AND care[tw] AND facilities[tw]) OR "ambulatory care facilities"[tw] OR "outpatients"[MeSH Terms] OR outpatients[tw] OR outpatient[tw] OR "hospitals"[MeSH Terms] OR hospitals[tw] OR hospital[tw] OR clinic[tw] OR clinics[tw] OR (medical[tw] AND center[tw]) OR "rehabilitation"[MeSH Terms] OR rehabilitation[tw] OR rehab[tw]) |
| --- | --- |
| #2 | Search ("self-help devices"[MeSH Terms] OR (self-help[tw] AND devices[tw]) OR "self-help devices"[tw] OR (self[tw] AND help[tw] AND devices[tw]) OR "self help devices"[tw] OR (assistive[tw] AND devices[tw]) OR "assistive devices"[tw] OR mechanical[tw] AND ("instrumentation"[Subheading] OR instrumentation[tw] OR devices[tw]) OR lift*[tw] OR hoist*[tw] OR "lift system"[tw] OR "lifting devices"[tw] OR (lifting[tw] AND device[tw]) OR "shower chair"[tw] OR "overhead lift"[tw] OR (overhead[tw] AND lift[tw]) OR "ceiling lift"[tw] OR (ceiling[tw] AND lift[tw]) OR ((mechanical[tw] OR adjustable[tw] OR electric*[tw]) AND bed[tw])) |
| #3 | Search ("low back pain"[MeSH Terms] OR (low[tw] AND back[tw] AND pain[tw]) OR "low back pain"[tw] OR LBP[tw] OR "back pain"[MeSH Terms] OR (back[tw] AND pain[tw]) OR "back pain"[tw] OR "back injuries"[MeSH Term] OR (back[tw] AND injuries[tw]) OR "back injuries"[tw] OR "shoulder pain"[MeSH Terms] OR (shoulder[tw] AND pain[tw]) OR "shoulder pain"[tw] OR "shoulder impingement syndrome"[MeSH Terms] OR (shoulder[tw] AND impingement[tw] AND syndrome[tw]) OR "shoulder impingement syndrome"[tw] OR "neck pain"[MeSH Terms] OR (neck[tw] AND pain[tw]) OR "neck pain"[tw] OR "neck injuries"[MeSH Terms] OR (neck[tw] AND injuries[tw]) OR "neck injuries"[tw] OR WMSD[tw] OR "work-related musculoskeletal disorder"[tw] OR "musculoskeletal disorder"[tw] OR (musculoskeletal[tw] AND disorder[tw]) OR "repetitive strain injury" OR (repetitive[tw] AND strain [tw] AND injury[tw]) OR "occupational overuse syndrome"[tw]) |
| #4 | Search (#1 AND #2 AND #3) |

**Table S2.** Excluded studies with reasons

**No Control Group (*n* = 33)**

1 Alamgir, H.; Yu, S.; Fast, C.; Hennessy, S.; Kidd, C.; Yassi, A. Efficiency of overhead ceiling lifts in reducing musculoskeletal injury among carers working in long-term care institutions. *Injury* **2008**, *39*, 570-577.

2 Caputo, F.; Lorusso, A.; Albano, I.; Bruno, S.; Sciannamblo, G.; Marinelli, G.; L'Abbate, N. Effect of an ergonomic intervention on low back injury trends in a large italian hospital. http://ovidsp.ovid.com/ovidweb.cgi?T=JS&PAGE=reference&D=emed7&NEWS=N&AN=2007058929 (Caputo, Lorusso, Albano, Bruno, Sciannamblo, L'Abbate) Sezione di Medicina del Lavoro B. Ramazzini, DIMIMP Universita di Bari, P.zza Giulio Cesare, 11, 70124 Bari, Italy),

3 Carta, A.; Parmigiani, F.; Roversi, A.; Rossato, R.; Milini, C.; Parrinello, G.; Apostoli, P.; Alessio, L.; Porru, S. Training in safer and healthier patient handling techniques. *British Journal of Nursing* **2010**, *19*, 576-582 577p.

4 Caspi, C.E.; Dennerlein, J.T.; Kenwood, C.; Stoddard, A.M.; Hopcia, K.; Hashimoto, D.; Sorensen, G. Results of a pilot intervention to improve health and safety for health care workers. *Journal of occupational and environmental medicine / American College of Occupational and Environmental Medicine* **2013**, *55*, 1449-1455.

5 Charney, W.; Simmons, B.; Lary, M.; Metz, S. Zero lift programs in small rural hospitals in washington state: Reducing back injuries among health care workers. *AAOHN Journal* **2006**, *54*, 355-358 354p.

6 Chhokar, R.; Engst, C.; Miller, A.; Robinson, D.; Tate, R.B.; Yassi, A. The three-year economic benefits of a ceiling lift intervention aimed to reduce healthcare worker injuries. *Applied ergonomics* **2005**, *36*, 223-229.

7 D'Orso, M.I.; Zoppini, L.; Dell'Acqua, M.; Toso, C.; Cesana, G.C. The adoption of electrical beds in hospital clinic units: Evaluation of the impact on workers' low back pathologies. http://ovidsp.ovid.com/ovidweb.cgi?T=JS&PAGE=reference&D=emed8&NEWS=N&AN=18409694 (D'Orso, Zoppini, Dell'Acqua, Toso, Cesana) Dipartimento di Medicina Clinica e Prevenzione, Universita di Milano Bicocca.),

8 Fekieta, R. Pre and post evaluation of a participatory ergonomics approach to promote usage of patient lifting equipment. University of Connecticut, 2007.

9 Fujishiro, K.; Weaver, J.L.; Heaney, C.A.; Hamrick, C.A.; Marras, W.S. The effect of ergonomic interventions in healthcare facilities on musculoskeletal disorders. *Am. J. Ind. Med.* **2005**, *48*, 338-347.

10 Garg, A.; Kapellusch, J.M. Long-term efficacy of an ergonomics program that includes patient-handling devices on reducing musculoskeletal injuries to nursing personnel. *Human factors* **2012**, *54*, 608-625.

11 Haglund, K.; Kyle, J.; Finkelstein, M. Pediatric safe patient handling. *Journal of Pediatric Nursing* **2010**, *25*, 98-107 110p.

12 Hinton, D.L.; Spencer, H.J.; Kortebein, P. Patient lift systems may not prevent injury claims in rehabilitation nurses and therapists. *PM & R : the journal of injury, function, and rehabilitation* **2009**, *1*, 287-288.

13 Hodgson, M.J.; Matz, M.W.; Nelson, A. Patient handling in the veterans health administration: Facilitating change in the health care industry. *Journal of occupational and environmental medicine / American College of Occupational and Environmental Medicine* **2013**, *55*, 1230-1237.

14 Huffman, G.M.; Crumrine, J.; Thompson, B.; Mobley, V.; Roth, K.; Roberts, C. On ships and safety: A journey of safe patient handling in pediatrics. Journal of Pediatric Nursing 2014, 29, 641-650 610p.

15 Hunter, B.; Branson, M.; Davenport, D. Saving costs, saving health care providers' backs, and creating a safe patient environment. *Nursing Economic$* **2010**, *28*, 130-134 135p.

16 Iwakiri, K.; Takahashi, M.; Sotoyama, M.; Liu, X.; Koda, S. Low back pain among workers in care facilities for the elderly after introducing welfare equipment. Sangyo eiseigaku zasshi = Journal of occupational health 2016, 58, 130-142.

17 Lahiri, S.; Latif, S.; Punnett, L.; ProCare Research, T. An economic analysis of a safe resident handling program in nursing homes. *Am. J. Ind. Med.* **2013**, *56*, 469-478.

18 Lipscomb, H.J.; Schoenfisch, A.L.; Myers, D.J.; Pompeii, L.A.; Dement, J.M. Evaluation of direct workers' compensation costs for musculoskeletal injuries surrounding interventions to reduce patient lifting. *Occup Environ Med* **2012**, *69*, 367-372.

19 Martin, P.J.; Harvey, J.T.; Culvenor, J.F.; Payne, W.R. Effect of a nurse back injury prevention intervention on the rate of injury compensation claims. *Journal of safety research* **2009**, *40*, 13-19.

20 Michaelis, M. [prevention of back disorders using a lifting tool-study of the intervention]. *Vorbeugung von Ruckenerkrankungen durch bodenlifter--eine Interventionsstudie.* **1997**, *51*, 18-21.

21 Nelson, A.; Matz, M.; Chen, F.; Siddharthan, K.; Lloyd, J.; Fragala, G. Development and evaluation of a multifaceted ergonomics program to prevent injuries associated with patient handling tasks. *International Journal of Nursing Studies* **2006**, *43*, 717-733 717p.

22 Olinski, C.; Norton, C.E. Implementation of a safe patient handling program in a multihospital health system from inception to sustainability: Successes over 8 years and ongoing challenges. Workplace Health Saf 2017, 65, 546-559.

23 Park, R.M.; Bushnell, P.T.; Bailer, A.J.; Collins, J.W.; Stayner, L.T. Impact of publicly sponsored interventions on musculoskeletal injury claims in nursing homes. *Am J Ind Med* **2009**, *52*, 683-697.

24 Passfield, J.; Marshall, E.; Adams, R. "No lift" patient handling policy implementation and staff injury rates in a public hospital. http://ovidsp.ovid.com/ovidweb.cgi?T=JS&PAGE=reference&D=emed6&NEWS=N&AN=2003141904 (Passfield) Townsville Hospital, 100 Angus Smith Drive, Douglas, QLD 4814, Australia),

25 Powell-Cope, G.; Toyinbo, P.; Patel, N.; Rugs, D.; Elnitsky, C.; Hahm, B.; Sutton, B.; Campbell, R.; Besterman-Dahan, K.; Matz, M.*, et al.* Effects of a national safe patient handling program on nursing injury incidence rates. *Journal of Nursing Administration* **2014**, *44*, 525-534 510p.

26 Przybysz, L.; Levin, P.F. Initial results of an evidence-based safe patient handling and mobility program to decrease hospital worker injuries. Workplace Health Saf 2017, 65, 83-88.

27 Ronald, L.A.; Yassi, A.; Spiegel, J.; Tate, R.B.; Tait, D.; Mozel, M.R. Effectiveness of installing overhead ceiling lifts: Reducing musculoskeletal injuries in an extended care hospital unit. *AAOHN Journal* **2002**, *50*, 120-127 128p.

28 Silverwood, S.; Haddock, M. Reduction of musculoskeletal injuries in intensive care nurses using ceiling-mounted patient lifts. *Dynamics* **2006**, *17*, 19-21 13p.

29 Stenger, K.; Montgomery, L.; Briesemeister, E. Creating a culture of change through implementation of a safe patient handling program [corrected] [published erratum appears in crit care nurs clin north am 2009 dec;21(4):595]. *Critical Care Nursing Clinics of North America* **2007**, *19*, 213-222 210p.

30 Stevens, L.; Rees, S.; Lamb, K.V.; Dalsing, D. Creating a culture of safety for safe patient handling. *Orthopaedic Nursing* **2013**, *32*, 155-166 112p.

31 Theis, J.L.; Finkelstein, M.J. Long-term effects of safe patient handling program on staff injuries. *Rehabilitation Nursing* **2014**, *39*, 26-35 10p.

32 Torri, P.; Liboni, D.; Milan, F.; Piccoli, R. An experience in management of risk due to manual lifting of patients in hospitals in the veneto region. http://ovidsp.ovid.com/ovidweb.cgi?T=JS&PAGE=reference&D=emed4&NEWS=N&AN=1999184667 (Torri, Liboni, Milan, Piccoli) Medico Competente Azienda ULSS 18, Rovigo, Regione Veneto, Italy),

33 Weinel, D. Successful implementation of ceiling-mounted lift systems. *Rehabilitation Nursing* **2008**, *33*, 63-87 65p.

**Not a research Study (*n* = 9)**

1. Anonymous. Lateral transfer device reduces back injuries that result from moving patients. Health care cost reengineering report 1998, 3, 157-158.
2. Anonymous. Ceiling hoists reduce risk of injury at tth. http://ovidsp.ovid.com/ovidweb.cgi?T=JS&PAGE=reference&D=emed10&NEWS=N&AN=22329042
3. Anonymous. Safe patient handling: New resources, information available. http://ovidsp.ovid.com/ovidweb.cgi?T=JS&PAGE=reference&D=emed10b&NEWS=N&AN=23444664
4. Bain, E. Using patient-handling equipment to reduce injuries. Massachusetts Nurse 2004, 75, 8-8 1p.
5. Cowell, R.; Shuttleworth, A. Equipment for moving and handling patients. Professional nurse (London, England) 1998, 14, 123-130.
6. de Castro, A.B. Actively preventing injury: Avoiding back injuries and other musculoskeletal disorders among nurses. The American journal of nursing 2004, 104, 104.
7. Evans, G. Healthcare workers remain at risk of patient handling injuries. Hospital Employee Health 2016, 35, 121-124.
8. Heiden, B. [preventing backaches with lifting aids: Still implemented too little]. Pflege Zeitschrift 2004, 57, 770-773.

9. Trautner, K. Workplace wisdom. Safe/no lifting culture can prevent injuries. Ohio nurses review 2010, 85, 18.

**Abstract only (*n* = 2)**

**1** Burgess, B.; Burgess, T.; Price, C.; Nichols, W.L. Use of ceiling mounted lifts with patient slings in the christiana emergency department considerably reduces the incidence of injury among emergency department and hospital personnel, who are required to lift and transfer patients. http://ovidsp.ovid.com/ovidweb.cgi?T=JS&PAGE=reference&D=emed10&NEWS=N&AN=70897453 (Burgess, Burgess, Price, Nichols) Christiana Care Health Services, Newark, DE, United States).

2 Garg, A.G.; Kapellusch, J.M.K. Ergonomic interventions in nursing facilities: Long-term effectiveness of a comprehensive program. http://ovidsp.ovid.com/ovidweb.cgi?T=JS&PAGE=reference&D=emed11&NEWS=N&AN=71745668 (Garg, Kapellusch) University of Wisconsin-Milwaukee, Milwaukee, United States)**.**

**No technical aids or technical aids not explicitly mentioned (*n* = 15)**

1. Alperovitch-Najenson, D.; Furas, R.; Kalichman, L. Factors affecting nursing staff compliance with the use of mechanized lifts: Paving the way towards a 'no manual lifting' policy. Journal of the Israeli Physical Therapy Society (JIPTS) 2011, 13, 31-31 31p.
2. Berthelette, D.; Leduc, N.; Bilodeau, H.; Durand, M.-J.; Faye, C. Evaluation of the implementation fidelity of an ergonomic training program designed to prevent back pain. Applied ergonomics 2012, 43, 239-245.
3. Brusco, N.K.; Taylor, N.; Stevens, J.A.; Butler, M.; Searle, A. Development of a manual handling programme for allied health.
4. Concordia, A.; Vaccari, A.; Casimirri, E.; Stendardo, M.; Ferroni, R.; Tonetto, G.; Nardini, M.; Boschetto, P. Reduction of injuries related to manual handling of patients: Two preventive methods used in five ferrara district hospitals. http://ovidsp.ovid.com/ovidweb.cgi?T=JS&PAGE=reference&D=emed10&NEWS=N&AN=23393853 (Concordia, Vaccari, Casimirri, Stendardo, Boschetto) Dipartimento di Medicina Clinica e Sperimentale, Sezione di Igiene e Medicina del Lavoro, Universita degli Studi di Ferrara, Italy),
5. Fanello, S.; Jousset, N.; Roquelaure, Y.; Chotard-Frampas, V.; Delbos, V. Evaluation of a training program for the prevention of lower back pain among hospital employees. Nursing & health sciences 2002, 4, 51-54.
6. Fragala, G.; Fragala, M. Improving the safety of patient turning and repositioning tasks for caregivers. Workplace health & safety 2014, 62, 268-273.
7. Iwakiri, K.; Matsudaira, K.; Ichikawa, K.; Takahashi, M. Effects of intervention program for systematic use of transfer equipment on care workers' low back pain in elderly care facilities. Sangyo eiseigaku zasshi = Journal of occupational health 2017, 59, 82-92.
8. Jaromi, M.; Kukla, A.; Szilagyi, B.; Simon-Ugron, A.; Bobaly, V.K.; Makai, A.; Linek, P.; Acs, P.; Leidecker, E. Back school programme for nurses has reduced low back pain levels: A randomised controlled trial. Journal of clinical nursing 2017.
9. Kozak, A.; Freitag, S.; Nienhaus, A. Evaluation of a training program to reduce stressful trunk postures in the nursing professions: A pilot study. Annals of work exposures and health 2017, 61, 22-32.
10. Rasmussen, C.D.; Holtermann, A.; Jorgensen, M.B.; Orberg, A.; Mortensen, O.S.; Sogaard, K. A multi-faceted workplace intervention targeting low back pain was effective for physical work demands and maladaptive pain behaviours, but not for work ability and sickness absence: Stepped wedge cluster randomised trial. Scandinavian journal of public health 2016, 44, 560-570.
11. Rasmussen, C.D.N.; Holtermann, A.; Mortensen, O.S.; Sogaard, K.; Jorgensen, M.B. Prevention of low back pain and its consequences among nurses' aides in elderly care: A stepped-wedge multi-faceted cluster-randomized controlled trial. BMC public health 2013, 13, 1088.
12. Risor, B.W.; Casper, S.D.; Andersen, L.L.; Sorensen, J. A multi-component patient-handling intervention improves attitudes and behaviors for safe patient handling and reduces aggression experienced by nursing staff: A controlled before-after study. Applied ergonomics 2017, 60, 74-82.
13. 1Shojaei, S.; Tavafian, S.S.; Jamshidi, A.R.; Wagner, J. A multidisciplinary workplace intervention for chronic low back pain among nursing assistants in iran. Asian spine journal 2017, 11, 419-426.
14. Warming, S.; Ebbehoj, N.E.; Wiese, N.; Larsen, L.H.; Duckert, J.; Tonnesen, H. Little effect of transfer technique instruction and physical fitness training in reducing low back pain among nurses: A cluster randomised intervention study. Ergonomics 2008, 51, 1530-1548.
15. Weber, M. Reducing undue back stress or injuries in rescue work and ambulatory transfer. http://ovidsp.ovid.com/ovidweb.cgi?T=JS&PAGE=reference&D=emed5&NEWS=N&AN=2000113863 (Weber) Arzt fur Allgemeinmedizin in Kronach, Amtsgerichtstrasse 3, 96317 Kronach, Germany).

**Population – not health care workers (*n* = 1)**

1 Nussbaum, M.A.; Torres, N. Effects of training in modifying working methods during common patient-handling activities. http://ovidsp.ovid.com/ovidweb.cgi?T=JS&PAGE=reference&D=emed5&
NEWS=N&AN=2000413109 (Nussbaum, Torres) Industrial and Systems Engineering, Virginia Polytechnic Institute and State University, 250 New Engineering Building (0118), Blacksburg, VA 24061, United States).

**Study Design (*n* = 20)**

1 Abedini, R.; Choobineh, A.R.; Hasanzadeh, J. Patient manual handling risk assessment among hospital nurses. Work 2015, 50, 669-675 667p.

2 Andersen, L.L.; Burdorf, A.; Fallentin, N.; Persson, R.; Jakobsen, M.D.; Mortensen, O.S.; Clausen, T.; Holtermann, A. Patient transfers and assistive devices: Prospective cohort study on the risk for occupational back injury among healthcare workers. Scandinavian journal of work, environment & health 2014, 40, 74-81.

3 Anyan, I.W.R.; Faraklas, I.; Morris, S.E.; Cochran, A. Overhead lift systems reduce back injuries amongst burn care providers. http://ovidsp.ovid.com/ovidweb.cgi?T=JS&PAGE=reference&D=emed11&NEWS=N&AN=71048445 ((Anyan III, Faraklas, Morris, Cochran) University of Utah, Salt Lake City, UT, United States),

4 Collins, J.W.; Wolf, L.; Bell, J.; Evanoff, B. An evaluation of a 'best practices' musculoskeletal injury prevention program in nursing homes. Injury Prevention 2004, 10, 206-211 206p.

5 Engkvist, I.-L. Evaluation of an intervention comprising a no lifting policy in australian hospitals. Applied ergonomics 2006, 37, 141-148.

6 Engkvist, I.L.; Hjelm, E.W.; Hagberg, M.; Menckel, E.; Ekenvall, L. Risk indicators for reported over-exertion back injuries among female nursing personnel. Epidemiology (Cambridge, Mass.) 2000, 11, 519-522.

7 Erich, J. Making the hard moves easier. What can reduce the injury risks of moving patients? http://ovidsp.ovid.com/ovidweb.cgi?T=JS&PAGE=reference&D=emed11&NEWS=N&AN=23638546

8 Garb, J.R.; Dockery, C.A. Reducing employee back injuries in the perioperative setting. AORN Journal 1995, 61, 1046-1052 1047p.

9 Gold, J.E.; Punnett, L.; Gore, R.J. Predictors of low back pain in nursing home workers after implementation of a safe resident handling programme. Occup Environ Med 2017, 74, 389-395.

10 Guthrie, P.F.; Westphal, L.; Dahlman, B.; Berg, M.; Behnam, K.; Ferrell, D. A patient lifting intervention for preventing the work-related injuries of nurses. Work 2004, 22, 79-88 10p.

11 Holtermann, A.; Clausen, T.; Jorgensen, M.B.; Aust, B.; Mortensen, O.S.; Burdorf, A.; Fallentin, N.; Andersen, L.L. Does rare use of assistive devices during patient handling increase the risk of low back pain? A prospective cohort study among female healthcare workers. Int Arch Occup Environ Health 2015, 88, 335-342.

12 Lee, S.-J.; Faucett, J.; Gillen, M.; Krause, N. Musculoskeletal pain among critical-care nurses by availability and use of patient lifting equipment: An analysis of cross-sectional survey data. International journal of nursing studies 2013, 50, 1648-1657.

13 Lee, S.-J.; Lee, J.H.; Gershon, R.R.M. Musculoskeletal symptoms in nurses in the early implementation phase of california's safe patient handling legislation. Research in Nursing & Health 2015, 38, 183-193 111p.

14 Lim, H.; Black, T.; Sarker, S.; Metcalfe, J. Long-term effect of multifactor transfer, lifting, and repositioning intervention program among health care workers. http://ovidsp.ovid.com/ovidweb.cgi?T=JS&PAGE=reference&D=emed10&NEWS=N&AN=71290979 ((Lim, Black, Sarker) University of Saskatchewan, Saskatoon, Saskatchewan, Canada),

15 Love, C. Lifting injury: A study of the occupational health perspective. Nursing standard (Royal College of Nursing (Great Britain) : 1987) 1997, 11, 33-38.

16 Ovayolu, O.; Ovayolu, N.; Genc, M.; Col-Araz, N. Frequency and severity of low back pain in nurses working in intensive care units and influential factors. http://www.pjms.com.pk/index.php/pjms/article/download/3455/1969

17 Owen, B.D. The lifting process and back injury in hospital nursing personnel. Western Journal of Nursing Research 1985, 7, 445-459 415p.

18 Samaei, S.E.; Mostafaee, M.; Jafarpoor, H.; Hosseinabadi, M.B. Effects of patient-handling and individual factors on the prevalence of low back pain among nursing personnel. Work 2017, 56, 551-561.

19 Smedley, J.; Egger, P.; Cooper, C.; Coggon, D. Manual handling activities and risk of low back pain in nurses. Occup Environ Med 1995, 52, 160-163.

20 Yeung, S.S.; Yuan, J. Low back pain among personal care workers in an old age home: Work-related and individual factors. AAOHN Journal 2011, 59, 345-353 349p.

**Outcome (*n* = 5)**

1 Craib, K.J.P.; Hackett, G.; Back, C.; Cvitkovich, Y.; Yassi, A. Injury rates, predictors of workplace injuries, and results of an intervention program among community health workers: Populations at risk across the lifespan: Empirical studies. http://ovidsp.ovid.com/ovidweb.cgi?T=JS&PAGE=reference&D=emed8&NEWS=N&AN=17319884 ((Craib, Hackett, Back, Cvitkovich, Yassi) Occupational Health and Safety Agency for Healthcare in BC, Vancouver, BC, Canada),

2 Daynard, D.; Yassi, A.; Cooper, J.E.; Tate, R.; Norman, R.; Wells, R. Biomechanical analysis of peak and cumulative spinal loads during simulated patient-handling activities: A substudy of a randomized controlled trial to prevent lift and transfer injury of health care workers. Applied ergonomics 2001, 32, 199-214.

3 Fragala, G. Reducing occupational risk to ambulatory caregivers. Workplace Health Saf 2016, 64, 414-419.

4 Lynch, R.M.; Freund, A. Short-term efficacy of back injury intervention project for patient care providers at one hospital. AIHAJ 2000, 61, 290-294 295p.

5 Schoenfisch, A.L.; Pompeii, L.A.; Myers, D.J.; James, T.; Yeung, Y.L.; Fricklas, E.; Pentico, M.; Lipscomb, H.J. Objective measures of adoption of patient lift and transfer devices to reduce nursing staff injuries in the hospital setting. http://ovidsp.ovid.com/ovidweb.cgi?T=JS&PAGE=reference&D=emed10&NEWS=N&AN=22068724 ((Schoenfisch, Myers, James, Lipscomb) Department of Community and Family Medicine, Division of Occupational and Environmental Medicine, Duke University Medical Center, Durham, NC, United States),

**Table S3.** Extracted results

| **Study, Year** | **Results** |
| --- | --- |
| Baldasseroni  2005 | 12-month prevalence low-back pain (number of episodes)  intervention group (n=106) /control group (n=176)  pre-intervention post-intervention  0 I: 73 (68.9%) C: 133 (75.6%) 0 I: 94 (88.7%) C: 139 (79.0%)  1-2 I: 24 (22.6%) C: 26 (14.8%) 1-2 I: 12 (11.3%) C: 27 (15.3%)  3-5 I: 7 (6.6%) C: 16 (9.1%) 3-5 I: NR C: 8 (4.5%)  >5 I: 2 (1.9%) C: 1 (0.6%) >5 I: NR C: 2 (1.1%) |
| Black 2011/ Lim 2011 | back injury claims (excluding neck) pre-intervention post-intervention  I: 112 (43.1%) I: 67 (44.4%)  C: 120 (63.2%) C: 90 (54.5%)  neck injury claims  pre-intervention post-intervention  I: 12 (4.6%) I: 10 (6.6%)  C: 16 (8.4%) C: 24 (14.5%)  shoulder injury claims  pre-intervention post-intervention  I: 31 (11.9%) I: 21 (13.9%)  C: 36 (18.9%) C: 4 (26.7%)  rate ratios musculoskeletal injuries  post vs. pre-intervention (ref.) RR_adj._= 0.69 (95%-CI 0.60–0.80) (This value was included in meta-analysis.)  intervention vs. control (ref.) RR_adj._= 1.42 (95%-CI 1.23–1.64)  *Poisson regression model included variables for group allocation, pre-/post-period, and hospital size.*  ---------  repeated back (including neck) injury claims  I: 86/413(21%) C: 75/231(32%)  repeated neck injury claims I: 3/41(7%) C: 4/58(7%)  repeated shoulder injury claims  I: 10/93(11%) C: 4/29(14%)  repeated musculoskeletal injury claims  intervention vs. control (ref.) OR_adj_.= 0.618 (95%-CI 0.27-0.81)  *Model adjusted for sex, age, occupation type, work department, hospital size, and body part injured.* |
| Dennerlein 2017 | 3-month prevalence low back pain  pre-intervention post-intervention adjusted* OR  I: 55.21% I: 49.99% 0.81 (95%-CI 0.63-1.04)  C: 53.66% C: 53.35% 0.99 (95%-CI 0.84-1.16)  3-month prevalence neck/shoulder pain  pre-intervention post-intervention adjusted* OR  I: 43.04% I: 40.56% 0.90 (95%-CI 0.70-1.16)  C: 42.43% C: 40.26% 0.91 (95%-CI 0.77-1.08)  3-week intensity of musculoskeletal pain (moderate pain severity >3)  pre-intervention post-intervention adjusted* OR  I: 44.84% I: 43.74% 0.96 (95%-CI 0.74-1.24)  C: 40.61% C: 42.11% 1.06 (95%-CI 0.91-1.25)  **ORs adjusted for random employee effect (a portion of the employees were sampled at baseline and follow-up)*  rate ratios injury claims post vs. pre-intervention (ref.)  intervention hospital  all injuries RR = 0.87 (95%-CI 0.76–1.00) (*This value was included in meta-analysis.)*  back RR = 0.792 (95%-CI 0.61-1.04)  neck/shoulder RR = 0.678 (95%-CI 0.46-1.00)  control hospital  all injuries RR = 0.889 (95%-CI 0.73–1.09)  back RR = 0.884 (95%-CI 0.54-1.45)  neck/shoulder RR = 0.713 (95%-CI 0.33-1.55) |
| Engst 2005 | raw number of “lifting & transferring related injuries” and “repositioning related injuries”.  lifting & transferring claims repositioning claims  pre-intervention post-intervention pre-intervention post-intervention  I: 5 I: 5 I: 7 I: 5 C: 5 C: 5 C: 4 C: 5 |
| Evanoff 2003 | musculoskeletal injury rate ratio (RR, 95%-CI), post-intervention vs. pre-intervention (ref.)  rates calculated as injuries per 100 full-time equivalents  all units combined RR = 0.82 (95%-CI 0.68-1.00)  acute care units RR = 0.86 (95%-CI 0.69-1.08)  LTC units RR = 0.71 (95%-CI 0.49-1.03)  “Adjusted risk ratios were calculated for workers in acute care units by estimating the expected number of injuries… in the post-intervention period based on temporal changes in rates among all other hospital workers at each facility....Because the intervention encompassed all nursing personnel at the LTC facilities, no adjustment of rates was possible.” Evanoff et al. 2003 |
| Fragala 2012 | raw number of injury claims 12-months pre-/post-intervention.  pre-intervention post-intervention  I: 4 I: 0  C: 2 C: 3 |
| Knibbe & Friele 1999 | 12-month back pain prevalence^a^ pre-intervention post-intervention  I: 98/132 (74%) I: 77/120 (64%) C: 138/223 (62%) C: 135/205 (66%) |
| Li 2004 | musculoskeletal injury rate ratio (RR, 95%-CI), post-intervention vs. pre-intervention (ref.)  rates calculated as injuries per 100 full-time equivalents  injuries RR_adj_ = 0.50 (95%-CI 0.20-1.26)  lost day injuries RR_adj_ = 0.35 (95%-CI 0.10-1.16)  “To control for potential temporal trends within the hospital regarding the overall injury rate, the injury rates of all non-intervention units were calculated using the same methods to compare changes during the pre-intervention and post-intervention time periods. These data were used to calculate an adjusted RR for the intervention units, assuming a decrease in expected injuries on the intervention units proportional to the decrease seen in the non-intervention units.” Li et al. 2004 |
| Miller 2006 | raw number of injury claims pre-/post intervention.  2 years pre-intervention 1 year pre-intervention post-intervention  I: 3 I: 2 I: 0  C: 8 C: 8 C: 3 |
| Smedley 2003 | 1-month prevalence low back pain^a^ pre-intervention: post-intervention:  I: 222/822 (27%) I: 248/827 (30%)  C: 90/333 (27%) C: 64/237 (27%) |
| Yassi 2001 | musculoskeletal injury rate  per 100 000 paid hr.  previous 3-year avg./ study year*).* arm A (control): 5.1 / 7.6  arm B (“safe lifting”): 6.3 / 5.3  arm C (“no strenuous lift”): 9.3 / 6.1  1-week prevalence of work-related low-back pain rating  t_0_=baseline, t_1_=6mo., t_2_=12mo.  arm A (control): arm B (“safe lifting”): arm C (“no strenuous lift”):  t_0_: 28.5 ±27.1 t_0_: 37.3 ± 27.7 t_0_: 34.0 ±27.5 t_1_: 37.2 ±31.4 t_1_: 36.2 ± 27.9 t_1_: 28.1 ±24.2  t_2_: 30.2 ±29.4 t_2_: 29.8 ± 24.2 t_2_: 31.7 ±27.6  arm B: 6 vs. 12 Mo.: p=0.016; 0 vs. 12 Mo.: p=0.012  no statistically significant change for arm A or arm C  changes in reported 1-week prevalence of low back rating at 6 months: 12 months:  arm A (control): 3.8 ± 27.5 1.0 ± 26.1  arm B (“safe lifting”): -0.6 ± 24.1 -6.5 ± 276  arm C (“no strenuous lift”): -5.2 ± 28.8 -3.3 ± 27.0  A vs. C (p=0.015) A vs. B (p=0.041)  Oswestry back disability scores  arm A (control): arm B (“safe lifting”): arm C (“no strenuous lift”):  t_0_: 5.4 ± 8.2 t_0_: 7.2 ± 8.8 t_0_: 5.7 ± 8.0 t_1_: 5.8 ± 9.3 t_1_: 7.2 ± 9.5 t_1_: 6.7 ± 9.0 t_2_: 6.4 ± 10.6 t_2_: 6.7 ± 8.7 t_2_: 5.4 ± 7.6  no statistically significant differences  1-week prevalence of work-related shoulder pain rating  arm A (control): arm B (“safe lifting”): arm C (“no strenuous lift”):  t_0_: 20.2 ±24.2 t_0_: 35.9 ± 29.7 t_0_: 26.5 ± 28.1  t_1_: 21.7 ±24.8 t_1_: 33.0 ± 29.4 t_1_: 21.6 ± 21.7  t_2_: 24.1±26.5 t_2_: 27.1 ± 24.9 t_2_: 24.2 ± 25.4  arm B: 6 vs. 12 Mo: p=0.034  arm B: 0 vs. 12 Mo.: p=0.012  no stat. significant change for arm A or arm C  changes in reported 1-week shoulder pain ratings at 6 months: 12 months:  arm A (control): 3.2 ± 23.8 4.4 ± 25.9  arm B: -1.3 ± 25.4 -6.6 ± 27.3  arm C: -4.9 ± 29.9 -2.3 ± 28.5  A vs. C (p=0.037) A vs. C (p=0.009)    DASH upper limb score (includes arm, shoulder and hand)  arm A (control): arm B: arm C:  t_0_: 5.2 ± 9.0 t_0_: 7.6 ± 8.9 t_0_: 6.6 ± 10.0 t_1_: 6.2 ± 11.7 t_1_: 8.0 ± 11.5 t_1_: 6.1 ± 9.9 t_2_: 6.3 ± 10.9 t_2_: 7.3 ± 10.9 t_2_: 5.5 ± 8.7  no statistically significant differences |

**I:** intervention group; **C:** control group; **RR:** Risk Ratio; **OR:** Odds Ratio; **95%-CI: 95%** confidence interval; **NR:** not reported
a) percentages reported, some numbers self-calculated from reported cases and response rates.

**Table S4a.** Risk of bias assessment for non-randomized studies with Downs & Black internal validity questions

|  | Baldassoroni 2005 | Black 2011/ Lim 2011 | Dennerlein 2017 | Engst 2005 | Evanoff 2003 | Fragala 2012 | Knibbe & Friele 1999 | Li 2004 | Miller 2006 | Smedley 2003 |
| --- | --- | --- | --- | --- | --- | --- | --- | --- | --- | --- |
| 14) blinding of subjects | HR | UR | UR | HR | UR | HR | LR | HR | UR | UR |
| 15) blinding of outcome assessors | UR | UR | UR | UR | UR | UR | UR | LR | UR | UR |
| 16) “data dredging” | LR | LR | LR | LR | LR | LR | LR | LR | LR | UR |
| 17) adjustment for different lengths of follow-up | HR | LR | LR | LR | HR | UR | LR | LR | LR | HR |
| 18) appropriate statistical tests | LR | LR | LR | HR | LR | HR | UR | LR | HR | LR |
| 19) intervention compliance | HR | LR | LR | LR | HR | UR | LR | HR | LR | UR |
| 20) outcome measurements accurate | LR | LR | LR | LR | LR | LR | LR | LR | LR | LR |
| 21) intervention & control groups from similar population | LR | LR | UR | LR | LR | LR | HR | LR | LR | UR |
| 22) intervention and control recruited over same period of time | UR | LR | LR | LR | LR | UR | LR | LR | LR | UR |
| 23) randomization | HR | | | | | | | | | |
| 24) allocation concealment | HR | | | | | | | | | |
| 25) adequate adjustment | HR | LR | UR | HR | HR | HR | HR | HR | HR | LR |
| 26) losses of patients taken into account | HR | UR | LR | LR | UR | UR | LR | UR | UR | HR |

**LR:** low risk; **HR:** high risk; **UR:** unclear risk

Was an attempt made to blind study subjects to the intervention they have received?

Was an attempt made to blind those measuring the main outcomes of the intervention?

If any of the results of the study were based on “data dredging”, was this made clear?

In trials and cohort studies, do the analyses adjust for different lengths of follow-up of patients, or in case-control studies, is the time period between the intervention and outcome the same for cases and controls?

Were the statistical tests used to assess the main outcomes appropriate?

Was compliance with the intervention/s reliable?

Were the main outcome measures used accurate (valid and reliable)?

Were the patients in different intervention groups (trials and cohort studies) or were the cases and controls (case-control studies) recruited from the same population?

Were study subjects in different intervention groups (trials and cohort studies) or were the cases and controls (case-control studies) recruited over the same period of time?

Were study subjects randomized to intervention groups?

Was the randomized intervention assignment concealed from both patients and health care staff until recruitment was complete and irrevocable?

Was there adequate adjustment for confounding in the analyses from which the main findings were drawn?

Were losses of patients to follow-up taken into account?

**Table S4b.** Risk of bias assessment for randomized studies

|  | **Yassi 2001** |
| --- | --- |
| Random sequence generation | UR |
| Allocation concealment | UR |
| Blinding of participants and researchers | HR |
| Blinding of outcome assessment | HR |
| Incomplete outcome data | UR |
| Selective reporting | UR |

**LR:** low risk; **HR:** high risk; **UR:** unclear risk
